# Recurrent hypoglycaemia in type-1 diabetes mellitus may unravel the association with Addison’s disease: a case report

**DOI:** 10.1186/1756-0500-7-634

**Published:** 2014-09-12

**Authors:** Stefano Passanisi, Tiziana Timpanaro, Donatella Lo Presti, Manuela Caruso-Nicoletti

**Affiliations:** Department of Medical and Pediatric Sciences, University of Catania; Azienda Ospedaliero Universitaria Policlinico-Vittorio Emanuele, Via Santa Sofia, n.78, 95123 Catania, Italy

**Keywords:** Type 1 diabetes mellitus, Addison’s disease, Hypoglycaemia, Screening

## Abstract

**Background:**

Primary adrenocortical insufficiency or Addison’s disease is caused by a progressive destruction of the adrenal cortex, resulting into a reduction of glucocorticoids, mineralocorticoids, and androgens. Autoimmune Addison’s disease is the most common etiological form, accounting for about 80% of all cases.

**Case presentation:**

We describe the case of a 16-year-old Caucasian boy affected by type-1 diabetes mellitus and autoimmune thyroiditis, who experienced recurrent hypoglycaemia as presenting symptom of Addison’s disease.

**Conclusions:**

Hypoglycaemia is not a common presenting feature of Addison’s disease, both in patients with type-1 diabetes mellitus and in non-diabetic patients. However, hypoglycaemia may occur in association with primary and secondary glucocorticoid deficiency as a result of an enhanced insulin sensitivity. Hypoglycaemia is the most common acute complication of insulin therapy in patients with type-1 diabetes mellitus. Addison’s disease has been described in approximately 0.5% of patients with type-1 diabetes mellitus, being more frequent in females and occurring in middle-aged patients. An association among type-1 diabetes mellitus, autoimmune thyroiditis, and Addison’s disease is found in the “Schmidt’s syndrome”, a rare disorder that may occur in the paediatric age. Our case suggests that the presence of Addison’s disease should be taken into consideration in patients with type-1 diabetes mellitus and frequent episodes of hypoglycaemia. We wish to highlight that there are no specific indications to screen for the association between Addison’s disease and type-1 diabetes mellitus, although an early diagnosis of Addison’s disease in diabetic patients would prevent the morbidity and potential mortality of this association.

## Background

Primary adrenocortical insufficiency, named Addison’s disease (AD), is caused by hypofunction/dysfunction of the adrenal cortex, resulting into a reduced production of glucocorticoids, mineralocorticoids and androgens associated with high levels of adreno-corticotrophic hormone (ACTH) and high plasma renin activity. Autoimmune AD is the most frequent form of AD in adult patients (about 80% of all cases), followed by post-tuberculosis AD (10–15% of cases). Vascular, neoplastic or rare genetic forms of AD account for 5% of cases [1]. Autoimmune AD is commonly associated with other autoimmune diseases, as occurs, for example, in types 1 and -2 autoimmune polyendocrine syndromes (APS) [2]. The prevalence of AD is 110–144 cases per million in developed countries. The rate is significantly increased in patients with type-1 diabetes mellitus (T1DM) [3, 4]. Cortisol deficiency increases insulin sensitivity resulting into an increased peripheral glucose utilisation, impaired gluconeogenesis, and decreased hepatic glucose output [5]. Hence, hypoglycaemia is expected to be a prominent and early feature of AD in diabetic patients. Although early diagnosis of AD in diabetes would prevent the morbidity and potential mortality of this association, there are not specific indications for the screening of AD in T1DM patients [6].

We describe the case of a young patient with T1DM and autoimmune thyroiditis with recurrent episodes of hypoglycaemia associated with the onset of AD.

## Case presentation

A 16-year-old Caucasian boy had been affected by T1DM since the age of 16 months. The diagnosis of insulin- dependent diabetes was confirmed by the following autoimmune profile: glutamic acid decarboxylase antibodies (GADA), 134 IU/ml; islet cell antibodies (ICA), 126 IU/ml; and antibodies directed towards protein tyrosine phosphatase-like protein (IA2-ab), 12 IU/ml. At the age of 4 years, autoimmune thyroiditis was diagnosed and treatment with levo-tiroxine was started. In the last 4 years, the patient had been treated with continuous subcutaneous insulin infusion (CSII), which, combined with an adequate lifestyle (balanced diet and daily physical activity), allowed to obtain an optimal glycaemic control.

During a follow-up visit, he reported sporadic episodes of hypoglycaemia in the preceding three months; one of these episodes was severe with loss of consciousness, and required admission to the hospital. At physical examination we observed: height = 180 cm; weight = 67 kg; body mass index (BMI) = 20.7 Kg/m^2^; blood pressure = 120/75 mmHg. General examination showed no abnormalities. Glycated haemoglobin was 7.5%. The patient was on a total daily insulin dose of 1.1 IU/Kg/day, with a percentage of basal insulin of 41%. We prescribed a reduction of the total insulin dose, and the insulin/carbohydrate ratio was reset, reassessing patient’s food diary.

The patient was revaluated three months later: other episodes of hypoglycaemia had occurred with one episode of severe hypoglycaemia characterized by seizure and treated with glucagon. At physical examination, body weight was unchanged; blood pressure was 105/55 mmHg, and glycated haemoglobin was significantly reduced (6.6%). General examination revealed no clinical signs indicative of any associated disease. The patient, spontaneously, had gradually reduced basal insulin (total 0.92 IU/kg/day) and had continued to show an adequate compliance with dietetic and therapeutic regimens. Both the patient and his parents denied alcohol abuse or improper lifestyle. Routine blood chemistry resulted negative, excluding the presence of liver and kidney diseases. Screening for celiac disease was also negative. Hence, we decided to further reduce the total dose of insulin.

At the following visit, the patient reported frequent hypoglycaemic episodes and marked asthenia. The clinical picture appeared clearly deteriorated: a significant weight loss was observed (weight 61 kg; BMI 18.7 Kg/m^2^). Downloaded data of the insulin pump indicated a further reduction of total insulin by more than 30% (0.75 IU/kg/day). Glycated haemoglobin value was 6.3%. At clinical examination, the patient presented scrotal hyperpigmentation and blood pressure = 80/50 mmHg.

Adrenal insufficiency was immediately suspected, and the patient was admitted to the hospital for further investigation. Blood tests showed: sodium, 129 mEq/L (136–145); potassium, 5.3 mEq/L (3.5-5.1); basal serum cortisol, 1.5 ng/ml; ACTH, 1274 pg/ml (<60). Blood cortisol levels in response to the ACTH test (1 μg i.v.) were the following: basal levels, 1.5 ng/ml; levels at 20’, 1.1 ng/ml; and levels at 30’ , 1.1 ng/ml. Adrenal antibodies could be detected.

The diagnosis of AD was confirmed and cortisone acetate (14 mg/m^2^ in three doses) was prescribed.

## Discussion

Hypoglycaemia is the most common acute complication in patients with T1DM. It is the main side effect of insulin therapy and an important limiting factor in the management of T1DM [7, 8]. Our case shows how the clinical approach to patients with T1DM and frequent hypoglycaemia should include the search for AD. Hypoglycaemia is not a common presenting feature of AD regardless of the presence of T1DM, although it has been associated with both primary and secondary glucocorticoid deficiency as a result of an enhanced insulin sensitivity [9]. Therefore, particularly in patients presenting abrupt reduction of insulin requirements and good compliance for dietetic and therapeutic regimens, the diagnosis of AD should be considered after excluding other potential causes of hypoglycaemia, such as excessive physical activity, significant reduction of carbohydrate intake, alcohol abuse, intestinal malabsorption, chronic renal failure, and liver disease. We did not measure insulin antibodies, which could be an additional cause of hypoglycaemia in our patient. Non-neutralizing insulin-binding antibodies induced by exogenous insulin may prolong the half-life of insulin in the circulation, thereby lowering insulin requirement [10, 11]. Insulin binding to antibodies has been considered as a risk factor for inexplicable hypoglycaemia in T1DM children [12].

Primary adrenocortical insufficiency or AD is caused by a progressive destruction of the adrenal cortex, resulting into a reduction of cortisol and aldosterone in both sexes, and a reduction of androgens in women [13]. The prevalence of AD is 110–144 cases per million in developed countries. Autoimmune AD is the most common etiological form of AD and may occur alone or be associated with other clinical, subclinical or potential autoimmune diseases, giving rise to various forms of autoimmune polyglandular syndrome (types 1, 2, or 4) [1]. AD is described in approximately 0.5% of patients with T1DM [3, 4]: it is more frequent in females and occurs in middle-aged patients, usually several years after the onset of T1DM [14].

T1DM, thyroiditis, and AD are associated in a clinical disorder known as Schmidt’s syndrome or type 2 autoimmune polyglandular syndrome (APS-II). The mean age at onset of Schmidt’s syndrome is about 30 years. Schmidt’s syndrome has an estimated prevalence of 1:200,000, and is very rare in the paediatric age [15]. However, an early onset of T1DM and thyroiditis in the first years of life could anticipate the disease [16].

To date, there are no national or international clear guidelines on the advisability of AD screening in paediatric diabetic patients.

Recent studies have demonstrated that 21 -hydroxylase autoantibodies (21OH-AA) have a low predictive value because the appearance of autoantibodies could anticipate the onset of the disease by many years. In addition, only 90% of T1DM patients with AD are 21OH-AA positive [17]. Moderately increased ACTH levels may be considered as an useful early indicator of AD, regardless of basal serum cortisol levels [6]. This data could identify those patients that should undergo a corticotrophin-stimulation test for the diagnosis of AD.

## Conclusions

The rarity of AD makes annual screening procedure uneconomic in patients with T1DM. The frequency of the screening procedure should depend on the characteristics of the patient. A diagnosis of T1DM in the first years of life and the coexistence of other autoimmune disorders, particularly autoimmune thyroiditis, should prompt to a closer inspection. Measurements of basal cortisol and ACTH levels should be immediately performed in patients with clinical suspect of AD. We wish to highlight that recurrent hypoglycaemia can be the only presenting feature of adrenal insufficiency, and, therefore, should encourage the examination of adrenal function. An early diagnosis of AD in T1DM patients is advisable to reduce morbidity and mortality in these patients.

## Consent

Written informed consent was obtained from the patient’s parents for publication of this Case Report. A copy of the written consent is available for review by the Editor-in-Chief of the journal.

## Abbreviations

AD: Addison’s disease
ACTH: Adreno-corticotrophic hormone
APS: Autoimmune polyendocrine syndrome
T1DM: Type 1 diabetes mellitus
GADA: Glutamic acid decarboxylase antibodies
ICA: Islet cell antibodies
IA-2: Antibodies directed towards protein tyrosine phosphatase-like protein
CSII: Continuous subcutaneous insulin infusion
BMI: Body mass index
APS-II: Type 2 autoimmune polyglandular syndrome
21OH-AA: 21-hydroxylase autoantibodies.
